# A Chitosan—Based Liposome Formulation Enhances the *In Vitro* Wound Healing Efficacy of Substance P Neuropeptide

**DOI:** 10.3390/pharmaceutics9040056

**Published:** 2017-12-06

**Authors:** Tamara Mengoni, Manuela Adrian, Susana Pereira, Beatriz Santos-Carballal, Mathias Kaiser, Francisco M. Goycoolea

**Affiliations:** 1 Institute of Plant Biology and Biotechnology (IBBP), Westfälische Wilhelms-Universität Münster, Schlossplatz 8, 48149 Münster, Germany; tamara.mengoni@gmail.com (T.M.); m_adri02@uni-muenster.de (M.A.); ssoar_01@uni-muenster.de (S.P.); mathiaskaiser@uni-muenster.de (M.K.); 2 ChiPrO GmbH, Anne-Conway-Strasse 1, 28359 Bremen, Germany; bcarballal@chipro.de or b_sant01@uni-muenster.de; 3 School of Food Science & Nutrition, University of Leeds, Leeds LS2 9JT, UK

**Keywords:** chitosan-coated liposomes, chitosan, wound healing, substance P, neuropeptide

## Abstract

Currently, there is considerable interest in developing innovative biodegradable nanoformulations for controlled administration of therapeutic proteins and peptides. Substance P (SP) is a neuropeptide of 11 amino acids that belongs to the tachykinins family and it plays an important role in wound healing. However, SP is easily degradable *in vivo* and has a very short half-life, so the use of chitosan-based nanocarriers could enhance its pharmaceutical properties. In light of the above, the aim of this work was to produce and characterize chitosan-coated liposomes loaded with SP (SP-CH-LP) as novel biomaterials with potential application in mucosal wound healing. The loaded system’s biophysical properties were characterized by dynamic light scattering with non-invasive back scattering (DLS-NIBS), mixed mode measurements and phase analysis light scattering (M3-PALS) and high performance liquid chromatography with ultraviolet/visible light detection (HPLC-UV/VIS). Then, the efficacy of the obtained nanoformulations was examined via proof-of-principle experiments using *in vitro* cell assays. These assays showed an increment on cell motility and proliferation after treatment with free and encapsulated neuropeptides. Additionally, the effect of SP on wound healing was enhanced by the entrapment on CH-LP. Overall, the amenability of chitosan-based nanomaterials to encapsulate peptides and proteins constitutes a promising approach towards potential novel therapies to treat difficult wounds.

## 1. Introduction

Research in novel wound healing therapies has gained traction because such therapies are relevant for treating life-threatening hemorrhages and severe burns and as well as for treating delayed acute and chronic wounds. Chronic wounds—generally correlated with diseases such as ischemia, diabetes mellitus, venous stasis disease, or pressure disorders—are known to exhibit impaired healing [1]. During the intricate process of wound healing, the injury site is associated with the migration and release of inflammatory cells, T cells, growth factors, cytokines, as well as stem and stromal cells. The repair course is divided into four different phases, namely coagulation, inflammation, proliferation and new tissue remodeling. Altogether, this leads to the closure of an open wound [2].

Further, several phases of the repair process such as inflammation and proliferation are influenced by the release of neuropeptides to the injury site. Among such peptides, substance P (SP) is noteworthy. SP, a neuropeptide of 11 amino acids [3], is a member of the tachykinin peptide family and is closely related to neuropeptide neurokinin A (NKA). SP has been documented to be released from the terminals of specific sensory nerve fibers and acts as a neurotransmitter and neurohormone in the body [4]. To perform its action, SP binds Neurokinin 1 receptor (NK1R), a G protein coupled receptor [4]. Existing *in vivo* studies agree that during tissue damage, the release of SP regulates and enhances wound healing via direct interaction with NK1R but also indirectly, by acting systemically [5,6]. Emerging research suggests that SP controls many pathways during the wound healing process; in fact, it has been reported that SP acts as vasodilator [7] and helps promote angiogenesis by increasing the recruitment of granulocytes [8] and the release of nitric oxide (NO) [9,10].

An increasing number of studies have revealed that SP also acts as a chemotactic agent at the injury site. Indeed, SP appears to intensify the release of cytokines and growth factors [11,12,13,14].

Several lines of evidence have also demonstrated that SP systemically induces mobilization of hematopoietic stem cells and stromal-like cells from the periphery to the injured tissues [15,16].

In the skin, SP has been related to the neurogenic inflammation, as it stimulates the regeneration of wounds via inducing the release of nerve growth factors [17], intensifying the migration of keratinocytes [14] and stimulating the proliferation of fibroblasts [18,19]. Improving our understanding on the action of SP and its effects on skin wound healing could be of relevance, for example, in the treatment of diabetic wounds. In fact, the neuropathy occurring in diabetics plays a role in the progression of impaired wound healing and it is known to be correlated to a diminished release of SP [1,6,12,20]. Of note, studies have also shown that SP can reverse diabetes in mice [21]. Thus, we anticipate that improved strategies for controlled delivery of SP could find potential application in treating diabetes-related chronic wounds and other disorders. 

Similar to other biological macromolecular drugs, *in vivo* SP is very susceptible to chemical and enzymatic degradation and it has a very short half-life, from seconds to minutes [13,22,23]. Enzymes that degrade SP are neutral endopeptidase (NEP) [24], SP degrading enzyme (SP-DE) [25] and angiotensin-converting enzyme (ACE) [26]; all of these enzymes cleave SP’s carboxyl-terminal active site and hence seriously compromise the bioavailability of SP. Although the therapeutic potential of SP is well recognized, studies have also revealed that SP can increase pain perception, thus compromising its therapeutic application. This caveat might be circumvented by a suitable formulation able to control the payload release. Indeed, a study found evidence that continuous infusion of SP into the striatum of rats alleviates pain by decreasing nociception rather than inducing it [27]. These reasons suggest that it may be desirable to develop a carrier system to enhance SP’s pharmacokinetic and pharmacodynamic parameters. 

Nanocarriers can protect the drug payload from degradation, increase intracellular absorption, provide a controlled and sustained drug release and maximize the location of the active compound on the site of application by a biotargeting mechanism. Currently, there is considerable interest in developing innovative formulations of nanocarriers for controlled administration of therapeutic proteins and peptides. Among such formulations, liposomes (LPs) are good candidates for the delivery of hydrophilic molecules such as charged and small molecules like peptides and non-Lipinsky macromolecules [28]. LPs are comprised mostly of phospholipids; as a result of their amphipathic nature, when dispersed in aqueous solutions LPs tend to spontaneously form a lipid bilayer that surrounds the aqueous core. Existing studies have shown that to optimize the characteristics and stability of LPs, they can be coated by a biopolymer such as chitosan [29,30,31,32,33]. 

Chitosan refers to a family of linear, cationic aminopolysaccharides produced by the partial deacetylation of chitin, which is isolated from crustacean exoskeletons, squid pen and fungi. They are biodegradable polymers composed of randomly distributed β(1→4)-linked *N*-acetyl-D-glucosamine (A) and D-glucosamine (D) sugar units and constitute a unique class of biopolymers [34,35]. Chitosan has received much attention due to its unique structural, physicochemical and biological properties, which include, among others, a polycationic character; solubility in aqueous medium under slightly acidic pH values; *in vivo* biodegradability via lysozymes and human chitinases [36]; mucoadhesivity; ability to complex genetic material *in vitro* and *in vivo*; antimicrobial activity; and wound healing capacity [37,38,39,40].

Chitosan has been known to coat the surface of negatively charged liposomes due to electrostatic interactions between the negatively charged phospholipids and the positive charges of primary amino groups of chitosan [30,41]. Many properties justify the use of chitosan as a biopolymer for coating LPs; in fact, chitosan coatings help increase the stability of LPs and prevent leakage from them. Further, this biopolymer coating increases the efficacy of the loaded drug, since it assures a sustained release, minimizes the drug release in undesired sites and increases the cellular uptake of the LPs by cells due to the chitosan coating’s positive charge [41]. In addition, the mucoadhesive properties of chitosan may mean that chitosan-coated LPs will exhibit beneficially prolonged residence times at the site of SP absorption due to increased contact of the carrier with the absorbing mucosa. Lastly, chitosan has antimicrobial activity against Gram-positive and Gram-negative bacteria [40,42,43], which highly necessary for promoting healing in the wound area. 

We believe that chitosan-coated liposomal formulations are a promising platform upon which to associate and deliver bioactive peptides and proteins. For this reason, in the present study we examined the delivery and efficacy of SP when encapsulated by a chitosan-based nanocarrier formulation. Our ultimate goal is to expand our understanding about how to increase the bioavailability of SP. To the best of our knowledge, no previous reports have documented the use of chitosan-based LPs for the delivery of SP with the goal of treating difficult chronic wounds such as diabetic wounds.

In light of the above, the aim of this study was to address the preparation, characterization and *in vitro* performance of SP- chitosan-coated LPs as a novel biomaterial with potential application in mucosal wound healing. Proof-of-principle of the *in vitro* efficacy and delivery of SP of the developed formulations were obtained in a human epidermal keratinocyte cell line (HaCaT).

## 2. Materials and Methods

All solvents were purchased from Sigma-Aldrich (Hamburg, Germany), Appli chem (Darmstadt, Germany), Carl Roth GmbH & Co. KG (Karlsruhe, Germany). 

A sample of biomedical-grade chitosan was obtained from Heppe Medical Chitosan Code Nr. HMC 70/5 (Batch No. 212-170614-01) (degree of acetylation was 12% as determined by ^1^H-NMR; molar mass 81.6 Kg·mol^−1^ as determined by SEC-HPLC with multidetection).

### 2.1. Preparation of Liposomes

Liposomes were prepared by the thin film hydration method described previously [36] with some modifications. Lecithin (Epikuron 145 V, Cargill, Minneapolis, MN, USA) and cholesterol (Sigma Aldrich, Saint Louis, MO, USA, CAS 57-88-5) were dissolved in 6 mL of chloroform/methanol (*v*/*v* = 1:1) at a concentration of 20 mg/mL and 3.3 mg/mL, respectively. The organic solvents were evaporated with a rotavapor (Büchi R-210 Büchi Labortechnik, GmbH, Essen, Germany) for 4 h at 150 mbar and T = 40 °C until the dry film lipid was formed. The hydration step was performed by resuspending the dry lipid film in 10 mL of water (pH = 4.5, 10 mM NaCl) stirring in a water bath (T = 40 °C) for >1 h. Finally, the liposome suspensions were sonicated in an ultrasonic bath (Elmasonic S 10 H, Elma, Singen, Germany) for 30 min. The uncoated liposomes were stored at 4 °C in the dark, ready for characterization and coating with chitosan. For the liposomes loaded with SP, the same protocol described above was followed but in addition, we added a constant quantity of substance P (ab120170) (Abcam, Cambridge, UK) to the water (pH = 4.5, 10 mM NaCl) before the hydration step.

### 2.2. Coating of Liposomes

Chitosan was previously dissolved in HCl (5% stoichiometric excess of equivalent D-glucosamine of chitosan) overnight at room temperature. In order to coat the liposomes with chitosan, 1 mL of chitosan with a concentration of 1 mg/mL was added dropwise to the same amount liposome volume under continuous magnetic stirring for 1 h. Finally, the liposomes were sonicated for 30 min with an ultrasonic bath (Elmasonic S 10 H, Elma, Singen, Germany) to produce uniform chitosan-coated liposomes. The final concentration of chitosan was set as 0.5 mg/mL.

### 2.3. Characterization of Chitosan-Coated Liposomes and Uncoated Liposomes

#### 2.3.1. Size and Zeta Potential Measurements

The uncoated and coated liposomes were characterized in terms of size and zeta potential using a Malvern Zetasizer Nano ZS (ZEN3600, Malvern Instruments, Worcester, UK) fitted with a red laser light beam (λ = 632.8 nm). The intensity size (hydrodynamic diameter) distributions of the formulations were determined by dynamic light scattering with non-invasive backscattering (DLS-NIBS) at 25 °C; the detection was at an angle of 173°. The ζ-potential was measured by phase analysis light scattering and mixed mode measurements phase analysis light scattering (M3-PALS) at 25 °C. The samples were diluted 1:100 in water for size measurements and 1:100 in 1 mM KCl for zeta potential measurements.

#### 2.3.2. Stability in Physiological Medium

To investigate the stability of the liposomes in physiological medium, an aliquot of SP-loaded liposomes, both coated and uncoated, were mixed with phosphate buffered saline (PBS) with the addition of salts (pH = 7.4). Size distribution and polydispersity index (PDI) were determined over time by DLS-NIBS using Malvern Zetasizer Nano ZS. Different time points were studied: 0 min, 30 min, 1 h, 2 h, 3 h and 24 h. The measurements were carried out at 37 °C in triplicate.

#### 2.3.3. High Performance Liquid Chromatography with Ultraviolet/Light Detection (HPLC/UV-VIS) Analysis

The quantification of SP was performed using high performance liquid chromatography (HPLC). The reversed-phase HPLC analyses were performed with a Jasco HPLC system (JASCO Labor-und Datentechnik GmbH; Gross-Umstadt; Germany) consisting of a 3-line degasser (DG-2080-53), a ternary gradient unit (LG-2080-02S), a semi-micro HPLC pump (PU-2085Plus), an autosampler (X-LC™ 3159AS), an intelligent column thermostat (CO-2060Plus) equipped with Aeris™ PEPTIDE XB-C18 reversed-phase column 150 mm × 2.1 mm with a particle size of 2.6 μm. (Phenomenex, Torrance, CA, USA). The mobile phase contained 70% of 0.1% (*v*/*v*) trifluoroacetic acid buffer and 30% of acetonitrile and had fixed flow rate of 0.2 mL/min at T = 40 °C in an isocratic gradient. 5-μL of sample were injected and the total run time was set at 4 min. SP was detected at 220 nm using a UV/VIS detector (X-L™ 3075UV). All experiments were performed in triplicate. The peak area (mV/min) of SP was determined automatically. The peak area corresponds to the concentration of SP in μg/mL.

#### 2.3.4. Encapsulation Efficiency

In order to determine the encapsulation efficiency (EE), Vivaspin2 centrifugal concentrators with a MWCO of 30,000 (Sartorius AG, Göttingen, Germany) was used to separate the non-encapsulated peptides from the liposomes. Aliquots of 1 mL of both coated and uncoated liposomes were placed in Vivaspin2 centrifugal concentrators and centrifuged two times for 30 min at 4000× *g* (Rotina 420 R, Hettich GmbH, Tuttlingen, Germany). Experiments were made in triplicate. The concentration of free SP that passed through the Vivaspin2 filter was determined using HPLC analysis with the conditions described above.

#### 2.3.5. Release Studies

An 800-μL aliquot of a chitosan-coated liposome formulation was transferred to a dialysis tube (Pur-A-LyzerTM Mini Dialysis Kit Mini 6000, MWCO = 6000 Da, Sigma-Aldrich, Munich, Germany) and placed in a glass beaker containing 9 mL of release medium composed of phosphate buffered saline with the addition of salts (pH = 7.4) previously equilibrated at 37 °C in an incubator. At determinate time point intervals, 100-μL aliquots were withdrawn from the release medium and the collected samples were replaced by an equal volume of fresh medium. The SP content of the aliquots was determined by HPLC-UV/VIS as described above. The experiments were performed in three independent replications. The kinetics of release of SP from the chitosan-coated liposomes was analyzed by fitting the data to the exponential equation known as the first-order drug release model. This model is commonly used to characterize the release profiles of drugs in delivery formulations [30].

### 2.4. Cell Culture

The human keratinocyte cell line HaCaT, obtained from the dermatological clinic at the University Hospital in Münster, Germany, were used to test the biological activities of SP. The cells were cultured using Dulbecco’s Modified Eagle Medium (DMEM) supplemented with 10% fetal bovine serum, 1% L-glutamine (200 mM) and 1% penicillin-streptomycin (10,000 units penicillin and 10,000 units streptomycin in 0.9% NaCl) in 75 cm^2^ flasks. The cultures were kept in an incubator set to 5% CO_2_ and 37 °C (Sanyo MCO-19AIC, Panasonic Biomedical Sales Europe BV, AZ Etten-Leur, The Netherlands). For the cell studies, all treatments were evaluated in cell culture medium with supplements and controls also consisted of cell culture medium with supplements.

#### 2.4.1. Cell Viability Assay

Cell viability was evaluated as a function of their relative metabolic activity using the 3-[4,5-dimethylthiazol-2-yl]-2,5 diphenyl tetrazolium bromide (MTT) test. Cells (1 × 10^4^/well) were seeded in 96-well plates in DMEM medium with supplements. After 24 h the culture medium was removed and the cells were rinsed twice with 100 μL/well PBS and the treatments were added (100 μL/well, *n* = 8 wells). DMEM with supplements was used as the negative control (100% viability) and a solution of 4% Triton X-100 in phosphate buffer saline was used as the positive control (0% survival). After 24 h of incubation, the treatments were removed and replaced with 100 μL of DMEM medium and 25 μL of MTT solution with a concentration of 5 mg/mL and the plate was incubated for 4 h at 37 °C. Finally, the culture medium and the MTT solution were removed and replaced with 100 μL DMSO (dimethyl sulfoxide) and the absorbance of the solubilized dye was measured at 570 nm with a microplate reader (Safire, Tecan AG, Austria) after orbital shaking at 300 rpm for 10 min. The result for each sample was calculated as the mean relative cell viability (*n* = 8) relative to the negative control.

#### 2.4.2. Wound Healing Assay

Cells in supplemented DMEM medium were seeded on culture-insert μ-Dishes (35 mm) (Ibidi GmbH, Planegg, Germany) at a density of 8.5 × 10^5^ cells/mL for wound healing assay. After 24 h, culture inserts were removed, cell monolayers with a cell-free gap of 500 µm were washed with PBS, and different treatments of free and encapsulated SP were applied to the cells. Cells treated with supplemented DMEM medium served as controls. Subsequently, the cells were incubated at 37 °C and at defined time points the dishes were placed under a phase-contrast microscope (Primo Vert, Carl-Zeiss Micro Imaging GmbH, Gottingen, Germany) equipped with a camera (Carl-Zeiss^®^, AxioCam ERc 5s, Göttingen, Germany) and images were acquired at 2× magnification with a computer-based microscopy imaging system (Axiovision^®^, Carl-Zeiss, Munich, Germany). To measure the wound healing activity of free and encapsulated SP, the closure of the gaps was monitoring for 23 h. Image analysis to quantify cell migration was made using Image J. For each image, the gap area was measured at certain time intervals and was compared to the initial gap area at time *t* = 0. Wound contraction (expressed as a percentage) was calculated from the following equation:

Wound contraction (%) = ((*W_t_*_0_ − *W_ti_*)/*W_t_*_0_) × 100

where, *W_t_*_0_ is the initial wound area at the outset of the experiment and *W_ti_* is the measured wound area at a given time interval.

#### 2.4.3. BrdU Colorimetric Assay

HaCaT cell proliferation activity was assessed by 5-Bromo-2′-deoxy-uridine (BrdU) colorimetric assay. The keratinocytes were plated in 96-well plates at a density of 1 × 10^4^ cells/well and cultured overnight. After 24 h, treatments of free and encapsulated SP in various concentrations were applied to the cells and cells were incubated for 24 h. DMEM plus supplements was used as a control. Thereafter, 10 μM of BrdU was added to each well. After 3 h, the labeling medium was removed, cells were fixed and the conjugated antibody (anti-BrdU-POD) was added. Incorporation of BrdU into the new DNA was determined using a cell proliferation kit (Roche) according to the manufacturer’s instructions. Experiments were performed in three independent replicates.

#### 2.4.4. Statistical Analysis

Statistical analysis was carried out using Prism v6.0c (GraphPad Software Inc., La Jolla, San Diego, CA, USA). All experiments were statistically analyzed using ANOVA followed by the Tukey multiple comparison test. Differences were considered statistically significant when *p* ≤ 0.05 (*), *p* ≤ 0.01 (**) or *p* ≤ 0.001 (***). All biological experiments were conducted at least in triplicate and technical replicates varied from 3 to 8. All the treatments were compared to the control cells, which were incubated with supplemented DMEM.

## 3. Results and Discussion

### 3.1. Preparation and Characterization of Liposomal Formulations

First, we examined the uncoated and chitosan-coated liposomes regarding their physical properties, stability in simulated physiological medium, association with the drug (SP) and *in vitro* release of SP. The size (Z-average diameter), polydispersity index, zeta potential and encapsulation efficiency of coated and uncoated liposomes are shown in Table 1. The uncoated liposomes (UN-LP) had an average size diameter of 151 ± 27 nm, while the coated systems (CH-LP), had a mean size of 243 ± 24 nm. The low PDI (∼0.2) is diagnostic of a monodisperse, monomodal size distribution and confirmed the uniform and reproducible size of the liposomes, in keeping with the overall small standard deviation values.

The size of uncoated and coated liposomes are in accordance with previous investigations, in which chitosans with a similar degree of acetylation as those used in our study have shown the ability to successfully coat liposomes. [44,45]. The presence of the chitosan coating is confirmed by the increase of the Z-average size (243 ± 24 nm) and by the inversion of the zeta potential from negative to positive values between the uncoated and coated systems. In fact, SP-loaded chitosan-coated liposomes (SP-CH-LP) showed a zeta potential around +30 ± 2.3 mV, while the SP-loaded uncoated liposomes (SP-UN-LP) had a zeta potential of −60 ± 1.3 mV. The negative zeta potential of the uncoated liposome surface can be explained by the inherent composition of soy bean lecithin that creates the bilayer of the liposomes. Soy bean lecithin Epikuron 145 is known to contain the neutral lipid phosphatidylcholine (around 50% of the content), fatty acids (approximately 10%), phosphatidylethanolamine (around 14%), phosphatidylinositol (max. 3%) and phosphatidic acid (max. 3%), per the manufacturer’s specifications. The mix of lipids and the presence of charged phospholipids like phosphatidylethanolamine and phosphatidic acid are responsible for uncoated liposomes’ negative zeta potential. Therefore, the main interaction at play between the positive amino groups of chitosan and oppositely charged lecithin phospholipids can be argued to be electrostatic in nature.

Loading SP into the uncoated liposomal formulations (SP-UN-LP) only minorly affects the Z-average size when compared with the uncoated unloaded systems (UN-LP), though it results in a decrease of the zeta potential (−49.0 ± 2.5 and −60.1 ± 1.3 mV, respectively). This result suggests that an electrostatic interaction is at play between SP and liposomes upon their formation in water. This is consistent with the high isoelettric point (pI) of SP (∼10.5); hence, having a net positive charge at the working conditions (water) enables SP to interact with the oppositely charged phospholipids at the liposome’s surface. This was an unexpected result, as SP was expected to be associated with the liposomes predominately within their aqueous core.

**Figure 1:**
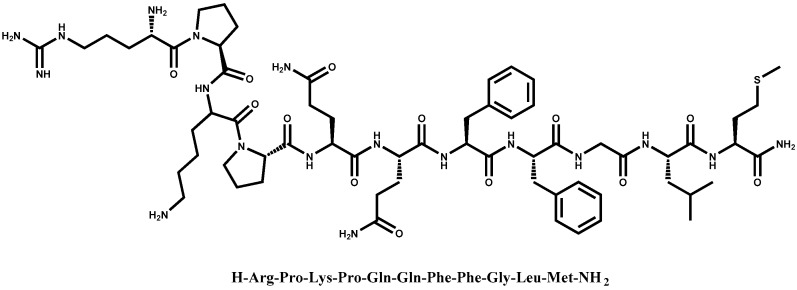
Chemical structure of substance P.

The encapsulation efficiency (EE) of SP in the liposomes was assessed by determining the concentration of free drug in solution after separating the liposomes using a Vivaspin ultrafiltration membrane cartridge and performing reverse phase HPLC analysis. The results displayed in Table 1 show that SP was encapsulated with efficiencies of 81.3 ± 6% and 66 ± 3.5% for the uncoated and chitosan-coated liposomes, respectively. These EE% values are considered high when compared to other water-soluble active pharmaceutical ingredients in liposomes [28]. This high EE% can be attributed to the entrapment of SP in the aqueous core of the liposomes and to the reinforced electrostatic interaction with negatively charged head group of lipid bilayer. Moreover, these interactions can occur both at the outer surface of the phospholipid bilayer and at the inner surface of the bilayer facing the core. Additionally, hydrophobic interactions should also be considered. Of note, SP has an amphiphilic nature due to the presence of aromatic residues at the C-terminus (Figure 1) and thus it might also be able to interact with the apolar fatty chains located in the middle of the phospholipid bilayer. As such, it is worth mentioning that some studies have revealed that lipids can induce a conformational change in SP [46]. Thus, the 15% lower EE% of SP observed in the chitosan-coated liposomes may be explained as an expected consequence of the positively charged chitosan and positively charged SP competing for the negatively charged phospholipids. This same effect has been observed in previous studies, showing a decrease in entrapment efficiency of positive charged compounds after liposomes were coated with chitosan [33].

**Figure 2:**
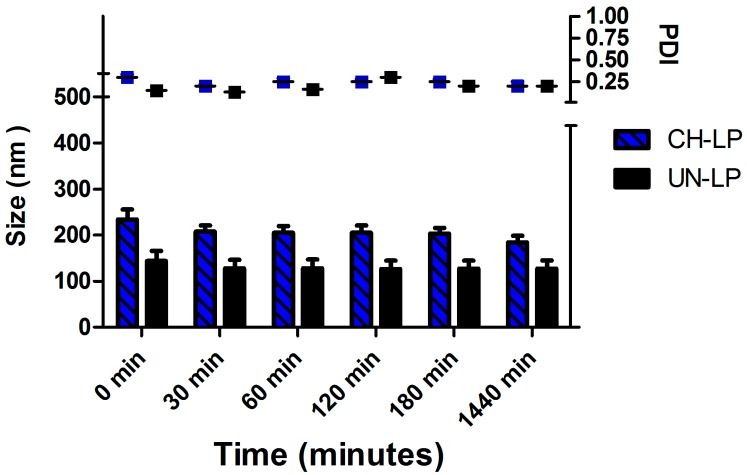
Stability assay of chitosan-coated liposomes (CH-LP) and uncoated liposomes (UN-LP) over 24 h in PBS medium.

Yet another important aspect to evaluate regarding the formulations was their stability upon incubation in PBS medium at 37 °C over 24 h (Figure 2), as these conditions are an approximate surrogate of the physiological milieu. We determined that the average diameter was ∼150 nm for the uncoated liposomes and ∼250 nm for the coated liposomes and the average size and the PDI did not increase over time, thus confirming that both systems remain stable against aggregation.

The results show that both systems are stable at 24 h, confirming that the coating with chitosan improves the stability [47]. Nevertheless, because the uncoated liposomes were easily destabilized in biological fluid (data not shown), we decided to assess the *in vitro* release profile of SP and perform cell studies using chitosan-coated liposome formulations. Indeed, the use of uncoated liposomes is limited by their thermodynamic instability and they are rarely administrated uncoated because they easily aggregate when stored and tend to degrade and leak materials upon administration [32,48].

### 3.2. In Vitro Release Profile

**Figure 3:**
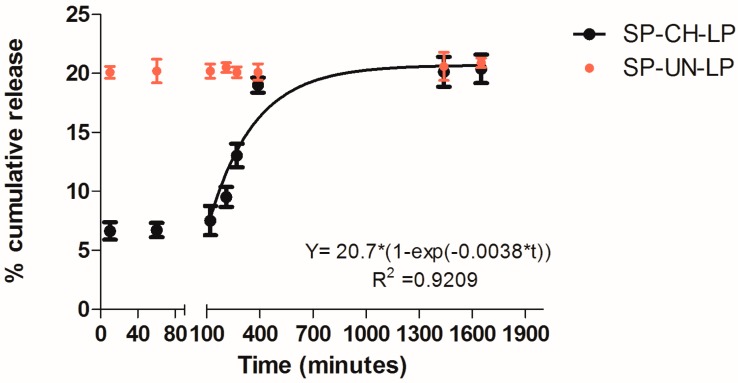
*In vitro* release of substance P from chitosan-coated and uncoated liposomes (as indicated in legend) in PBS (pH 7.4; 37 °C; *n* = 3).

*In vitro* SP release from chitosan-coated liposomes (CH-LP) and uncoated liposomes (UN-LP) was performed in phosphate buffered saline (PBS) at pH 7.4; 37 °C to simulate physiological conditions over 24 h (Figure 3). The chitosan-coated liposomes showed a two-stage profile release, as indicated by virtually no release during the first ∼100 min, followed by an exponential increase in SP release after this time. After 24 h, the liposomes released in total 20% of the drug payload; then it reached a plateau. The release profile during the second stage was fitted to a first-order kinetic mechanism of release (R^2^ ≥ 0.92), thus suggesting a promising model for sustained drug release, in accordance with previous release tests from chitosan-coated liposomes [30,45].

As an interpretation of the unique *in vitro* SP release behavior from coated liposomes, we suggest that the slowed initial release period is due to the strong binding of the drug at the liposome lipid bilayer membrane, which is also reinforced by the chitosan coating, as evidenced by the increase in particle size by >∼100 nm (Table 1).

This effect seems to reduce the permeability of the SP payload from the liposomal core during the initial release stage. The duration of this stage may be directly dictated by the gradual dissociation of the chitosan-phospholipid complex in PBS buffer.

It is yet to be determined whether the retardation period can be fine-tuned in future studies, for example, by using chitosans of varying degrees of acetylation or molar mass, or by constructing a layer-by-layer self-assembled coating (e.g., comprising chitosan and alginate). 

Further, also the release of SP from uncoated liposomes was registered. The naked liposomes release 20% of SP content already after 10 min. Thereafter, it is showed that chitosan-coated liposomes release SP in a retarded and slower manner as compared to the non-coated liposomes. This is the evidence that CH-LP create a prolonged and sustained release of SP and they would be particularly suitable for passively targeting.

Targeting the SP-CH-LP to inflammatory sites would allow for the exploitation of two known mechanisms: the enhanced permeability retention (EPR) effect and the phospholipase A2 (PLA2)-triggered pathway. The EPR effect occurs not only in tumors but also during inflammation in the wound healing process [49]. Additionally, some inflammatory sites show higher expression of secretory phospholipase A2 (PLA2) [50]; consequently, PLA2 could potentially trigger an even further enhanced release of a loaded compound from liposomes *in vivo*. Moreover, the slowed release profile of SP-CH-LP combined with passive targeting may allow for the development of advanced drug delivery formulations with space-time “programmed release” capacities. Such formulations could be attractive in wound healing therapy, as they could decrease the release of SP in non-target tissues, thus avoiding undesired effects provoked by the uncontrolled binding of SP with NK1R receptors and by the uptake of liposomes by cells not involved in wound healing.

### 3.3. In Vitro Cytotoxicity

We studied the influence of different concentrations of SP, both free and encapsulated in the liposomes, on the viability of HaCaT cells, using the 3-(4,5-dimethylthiazol-2-yl)-2,5-diphenyltetrazolium bromide (MTT) assay. The MTT assay is a colorimetric assay used to assess the cell viability given by mitochondrial metabolic activity. NAD(P)H-dependent cellular oxidoreductase enzymes can reduce the MTT dye to formazan, which has a purple color. The absorbance of the formazan is an indicator of the cell’s metabolic activity. Treatments were applied using free and encapsulated SP at three concentrations (1 × 10^−5^ M, 2.2 × 10^−5^ M and 4.4 × 10^−5^ M) and then the effects of the treatments on cell toxicity were assessed after an incubation period of 24 h. The reasons HaCaT cells (human epidermal keratinocyte cell line) were chosen for this assay were two-fold: first, the ideal route of administration of the developed liposomal formulations would be the skin, second, the HaCaT cell line was recently shown to express NK1R receptors [51].

**Figure 4:**
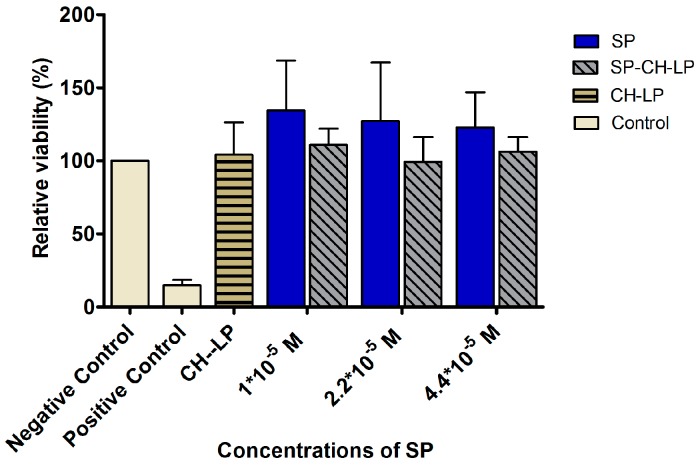
Effect of treatment with varying concentrations of free and liposome-encapsulated substance P on relative cell viability (metabolic competence) in HaCaT cells, as measured by MTT over 24 h (data represent the mean average ± standard deviation, *n* = 8). Triton X and cell culture media with supplements were used as positive and negative controls, respectively.

Figure 4 summarizes the results of the relative cell viability of HaCaT cells treated with free SP, with SP-loaded chitosan-coated liposomes and with positive (Triton X) and negative (DMEM plus supplements) controls. In all treatments, we observed a relative cell viability of ∼100%, thus indicating that the free SP or the liposomally-loaded SP formulations showed no toxic effects on keratinocytes at any of the tested concentrations. It was interesting to notice an increase of viability over 100% for free SP, indicating that the HaCaT cells increased their metabolic activity in the presence of SP.

SP naturally occurs throughout the human body and data indicate that the endogenous level of SP is ∼105.9 pmol/g in the spinal cord [52] and 0.05 pmol/mL in cerebrospinal fluid [53]. Since these concentrations are much lower than the doses used in our study, our results are promising in that they show these higher levels of SP do not result in toxicity towards HaCaT cells.

The choice of the SP concentration lower than 1 × 10^−5^ M used in this work, was motivated by the doses used in previous studies that resulted in SP successfully enhancing wound healing *in vivo* and *in vitro* [11,14,15]. SP treatments higher than 1 × 10^−5^ M are reasonable because liposomes can act as a reservoir system to guarantee sustained perfusion of SP over a long period while avoiding fluctuations. We thus reasoned that even at high SP concentrations, liposomes would be able to help maintain the minimum effective therapeutic level and prevent SP overdose. Another study also assessed the toxicity of free and nanoparticle-encapsulated SP on PC-12 cells; their results showed a bell-shaped toxicity curve, revealing an increase in cell viability when SP concentrations were lower than 1 × 10^−7^ M and a decrease in viability when SP concentrations were 1 × 10^−6^ M or 1 × 10^−5^ [54].

### 3.4. In Vitro Wound Healing Effect

**Figure 5:**
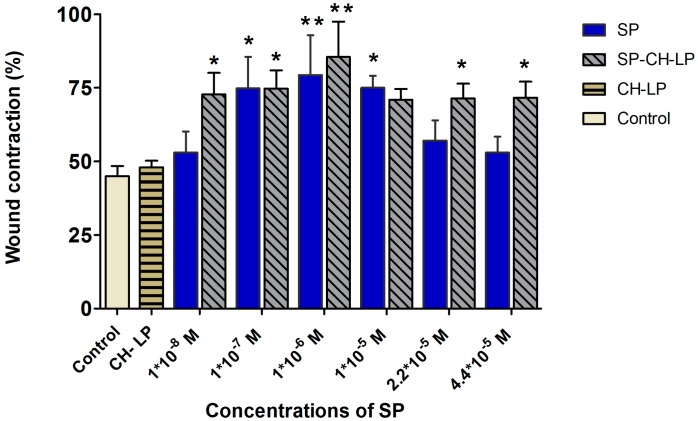
Effect of treatment with varying concentrations of free and liposome-encapsulated substance P on relative wound healing in HaCaT cells, as measured by Ibidi^®^ silicone insert *in vitro* wound healing assay over 23 h (data represent the mean average ± standard deviation, *n* = 3 to 6; cell culture media (DMEM) with supplements were used as control; stars on bars represent significant differences with respect to control by ANOVA followed by the Tukey multiple comparison test. * *p* < 0.05, ** *p* < 0.001).

The *in vitro* wound healing assay was also performed on HaCaT cells to evaluate the effects of the neuropeptide on cell migration. In the interest of reducing as much as possible the experimental errors, we used Ibidi^®^ silicone inserts with fixed 500-µm wide “wounds.” After inserts were removed, a reproducible and well-defined physical gap was created between the cells, which was used as a surrogate for an inflicted wound; cell migration was then tracked with the aid of a light microscope and image analysis software for 23 h. The cells were treated with either low SP concentrations of 1 × 10^−8^, 1 × 10^−7^ and 1 × 10^−6^ M, or high SP concentrations of 1 × 10^−5^, 2.2 × 10^−5^ and 4.4 × 10^−5^ M for both free SP and SP-loaded CH-LP. The wound reparation was evaluated for 23 h, during which we monitored the relative reduction of wound area with respect to time zero (Figure 5). We set 23 h as the end point because this was the last time point before cells become fully confluent (at 24 h) and start proliferating. Hence, the degree of the gap closure provides information about the cell motility. Exemplary pictures of time course wound healing are illustrated in Figure S1 (Supplementary Materials).

Close inspection reveals that at 23 h, the cells treated with 1 × 10^−6^ M free SP closed their gap by 82 ± 4.6%, a value significantly (*p* ≤ 0.01) greater than that registered for the untreated cells (45 ± 2.36%). The results showed a remarkable effect on the gap (wound) closure in a dose-dependent manner at the low SP concentrations of 1 × 10^−8^, 1 × 10^−7^ and 1 × 10^−6^ M with wound reparation of 52 ± 7.2%, 75 ± 10.7% (*p* ≤ 0.05) and 79.3 ± 13.6% (*p* ≤ 0.01), respectively. Free SP at 1 × 10^−8^ M turns out to be under the minimum effective concentration, since it affords similar results as the control. Regarding the treatments with free SP at high concentrations, a dose of 1 × 10^−5^ M led to significant wound closure of 71 ± 3.6% (*p* ≤ 0.05); by contrast, at higher doses (2.2 × 10^−5^ and 4.4 × 10^−5^ M) the wound distances decreased to 56 ± 7% and 53 ± 5.4%, respectively. Our studies are in accordance with previous evidence showing that free SP enhances keratinocyte migration *in vitro* models [14] and our study additionally confirms that free SP strongly influences the migration of keratinocytes (i.e., wound repair activity) described by a “Goldilocks” (i.e., bell-shaped) dose-dependent effect. The maximal wound closure effect was attained at an intermediate dose of 1 × 10^−6^ M SP; below or above this dose, lower activity was registered. This effect may arise because of saturation of the receptor up to a dose of 1 × 10^−5^ M of SP, while at greater concentrations of SP, the NK1R receptor may undergo desensitization, internalization, uncoupling, or depletion of the second messenger. The homologous desensitization of NK1R, which involves a slowly reversible downregulation or sequestration of SP-binding sites thus resulting in a negative feedback system, is typical of G-protein coupled receptors as it is reported in many studies [4,7,55,56,57,58]. 

The “Goldilocks” concentration-response curves observed for the HaCaT cell migration has been found also in another study, in which a supra-physiological concentration of free SP led to a saturation effect, resulting in SP having a decreased biological effect [11].

Our results suggest that a therapeutic window for free SP may be from 1 × 10^−7^ M to a maximum of 1 × 10^−5^ M.

Because liposomes can influence the biological effect of SP on wound healing (as previously mentioned), SP encapsulated in chitosan-coated liposomes at doses of 1 × 10^−8^, 1 × 10^−7^ and 1 × 10^−6^ M resulted in wound closures of 72 ± 7.3% (*p* ≤ 0.05), 75 ± 6.3% (*p* ≤ 0.05) and 85.5 ± 12% (*p* ≤ 0.01), respectively, with no apparent dose-dependent effect.

On average, the cell migration increased by ∼25% upon treatment with SP-CH-LP with respect to the control. Interestingly, at the larger doses of 2.2 × 10^−5^ and 4.4 × 10^−5^ M, SP-loaded CH-LP resulted in wound closures of 71 ± 5% and 71.6 ± 5.5%, respectively, very similar to those observed at lower doses. Further, at each given concentration, the effect on the wound healing of SP encapsulated in chitosan-coated liposomes was significantly higher compared to the free SP, particularly for the lowest (1 × 10^−8^ M) and the highest (2.2 × 10^−5^ and 4.4 × 10^−5^ M) doses. 

Based on this evidence, we can conclude that SP-CH-LP enlarges the therapeutic concentration window compared to free SP and that the overall efficacy of SP is significantly enhanced when it is loaded in chitosan-coated liposomes. The unloaded liposomes were also tested to eliminate the hypothesis that the wound healing effect was caused by chitosan. The results show that the unloaded chitosan-coated liposomes do not possess the ability to enhance wound healing. Hence, the observed effects can be attributed to the effect of SP being encapsulated in chitosan-coated liposomes.

The increase in wound repair observed using the SP-CH-LP formulation confirms the assumption that the loading SP into nanocarriers protects it from being degraded and influences its cellular uptake via endocytosis, bringing about the activation of yet to be elucidated wound repair pathways. Although these pathways have not been explored yet, we speculate that the observed effect on wound healing may involve more than just the agonist effect on the NK1 receptor.

**Figure 6:**
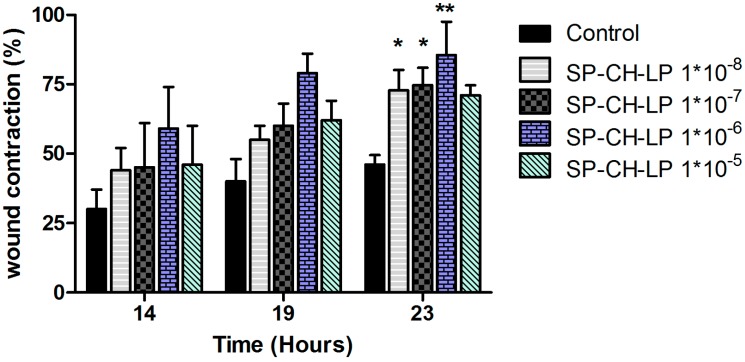
Effect of treatment with varying concentrations of liposome-encapsulated substance P on relative wound healing in HaCaT cells registered at varying times, as measured by Ibidi^®^ *in vitro* wound healing assay over 23 h. (Data represent the mean average ± standard deviation, *n* = 3 to 6; cell culture media (DMEM) with supplements were used as control; stars on bars represent significant differences respect to control by ANOVA with the Tukey multiple comparison test * *p* < 0.05, ** *p* < 0.001).

If this is the case, this may explain the lack of an evident dose-dependent effect and the efficacy of the highest SP concentration, which would otherwise lead to desensitization of the NK1 receptor and produce an inhibitory effect, as observed for free SP. To shed light onto the possible biological effects produced by the SP-loaded liposomes, we carried out time course wound healing assays on SP-CH-LP at varying doses at time points of 14, 19 and 23 h (Figure 6).

From Figure 6, we see a dose-dependent effect at 14 and 19 h, while at 23 h the wound contraction is markedly increased, reaching the highest significant (*p* < 0.05) effect for most of the SP concentrations used. The dose-dependent effect for the SP-CH-LP at 14 and 19 h indicates that different pathways are involved during the migration of keratinocytes. Indeed, the liposomes may be more effective on pathways that occur toward the early stages of *in vitro* wound healing rather than the later stages. As discussed above, SP does promote wound healing through many pathways, such as via vasodilatation, angiogenesis, chemotactic activity and activation of immune cells, cytokines and growth factors. Therefore, we must take into account that *in vitro* experiments cannot mimic all of these effects, nor those involved in such a dynamic and complex process as wound healing. So, the observed results are probably the consequence of several regulatory pathways within cells that are mediated by SP, such as cytoskeleton reorganization and/or expression of focal adhesion proteins.

### 3.5. SP Proliferation Effect on HaCaT Cells

**Figure 7:**
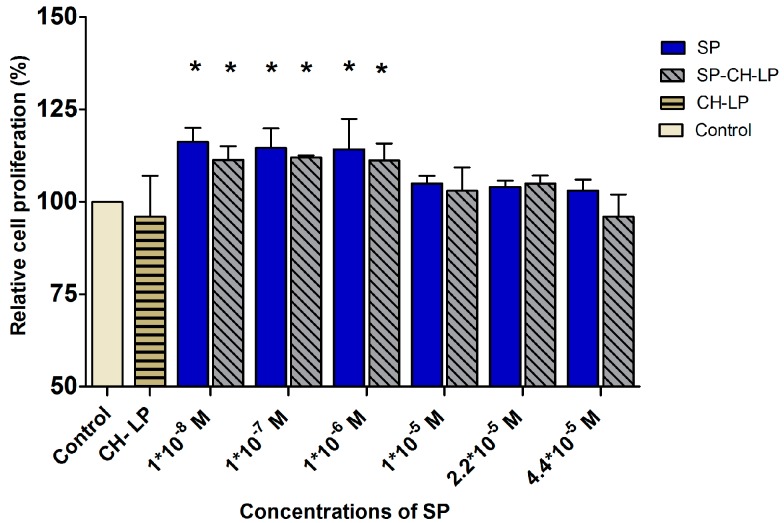
Effect of varying concentrations of free and liposome-encapsulated substance P on relative cell proliferation in HaCaT cells, as measured by BrdU colorimetric assay over 24 h. (Data represent the mean average ± standard deviation, of three independent biological experiments.) Cell culture medium (DMEM) with supplements was used as control; stars on bars represent significant differences with respect to the control by ANOVA with the Tukey multiple comparison test (* *p* < 0.05).

Cell proliferation activity is usually quantified by monitoring the nucleosides that get integrated into the cellular genetic material during proliferation. The most well-known assay is based on bromodeoxyuridine (BrdU), an analogue for thymidine. BrdU replaces thymidine in the newly synthesized genetic material and with the help of a secondary antibody, a colorimetric readout can be used to quantify the amount of integrated BrdU as an indicator of cell proliferation. This assay was used to monitor the effect of free and encapsulated SP on cell proliferation 24 h after treatment (Figure 7). The experiment was performed in presence of cell culture medium supplemented with FBS and the control refers to the cell culture medium, also supplemented but without SP. For the concentrations of 1 × 10^−8^, 1 × 10^−7^ and 1 × 10^−6^ M, the free and encapsulated SP stimulated cell proliferation in a manner not significantly different (within 15%) than in the untreated controls. This finding conflicts with earlier studies, which observed that SP can promote proliferation of spinal cord-derived neural stem cells [59], bone marrow stromal cells, [15] and fibroblasts [18]. Further, a previous study showed that SP stimulated cellular proliferation of keratinocytes *in vitro* in a concentration-dependent manner when SP was added in the concentration range of 1 × 10^−10^ to 1 × 10^−7^ M [11]. One additional study suggested that primarily CGRP, and to a lesser extent SP, influenced epidermal thickness and proliferation of keratinocytes in innervated skin models [60]. Besides that, the literature is lack of studies regarding the effect of free SP on the motility and proliferation of keratinocytes *in vitro* models, on the other hand data supply several evidences on wound closure activity by increasing of cell motility and proliferation after topically treatments with SP on skin wound *in vivo* or *ex vivo* [5,6,12,61,62].

## 4. Conclusions

In the present study, a new chitosan-coated liposome carrier was developed for SP delivery and we anticipate that this formulation may work in synergy with SP for wound healing applications. We found that the chitosan coating improved the stability of liposomes in physiological conditions and modified the release of SP in a “programmable” way. We provide *in vitro* proof of concept of the wound healing efficacy of free SP and SP encapsulated in liposomes. We found that when SP is encapsulated within the developed liposomal formulation, the minimum effective level of SP is decreased and, upon administration, the SP-encapsulated chitosan-coated liposome offers a wider therapeutic window and better efficacy than free SP, probably due to the longer and sustained release of SP. Additionally, we hypothesized that new wound repair pathways that are not yet elucidated could be involved in the strong effect of SP encapsulated in liposomes. Finally, we suggest that a potential application for SP-loaded liposome formulations is diabetic wounds (e.g., topical and subcutaneous application in cream and/or gel formulations).

## Figures and Tables

**Table 1:** Physicochemical properties of coated (CH-LP) and uncoated liposomes (UN-LP) and the corresponding SP-loaded liposomes (SP-CH-LP and SP-UN-LP) (*n* = 3).

Formulations	Size (d.,nm) ^1^	PdI ^1^	ζ (mV) ^2^	EE (%) ^3^
SP-UN-LP	151 ± 27	0.2 ± 0.06	−49 ± 2.5	81.3 ± 6
UN-LP	104 ± 9	0.2 ± 0.01	−60 ± 1.3	
SP-CH-LP	243 ± 24	0.3 ± 0.1	+32 ± 1.0	66 ± 3.5
CH-LP	247 ± 26	0.2 ± 0.06	+30 ± 2.3	

^1^ By DLS-NIBS; ^2^ By M3-PALS; ^3^ BY HPLC-UV-VIS.

## Supplementary Materials

The following are available online at www.mdpi.com/1999-4923/9/4/56/s1, Figure S1: Exemplary pictures of time course wound healing. (**A**) shows wound progression without treatments (control), while (**B**) and (**C**) illustrate the wound progression after treatment with free SP 1 × 10^−5^ M and SP-CH-LP 1 × 10^−5^ M, respectively.

## References

[1] Guo S., DiPietro L.A. (2010). Factors affecting wound healing. J. Dent. Res..

[2] Werner S., Grose R. (2003). Regulation of wound healing by growth factors and cytokines. Physiol. Rev..

[3] Tregear G.W., Niall H.D., Potts J.T., Leeman S.E., Chang M.M. (1971). Synthesis of substance P. Nat. New Biol..

[4] Steinhoff M.S., von Mentzer B., Geppetti P., Pothoulakis C., Bunnett N.W. (2014). Tachykinins and their receptors: contributions to physiological control and the mechanisms of disease. Physiol. Rev..

[5] Delgado A.V., McManus A.T., Chambers J.P. (2005). Exogenous administration of substance P enhances wound healing in a novel skin-injury model. Exp. Biol. Med. (Maywood).

[6] Kant V., Kumar D., Kumar D., Prasad R., Gopal A., Pathak N.N., Kumar P., Tandan S.K. (2015). Topical application of substance P promotes wound healing in streptozotocin-induced diabetic rats. Cytokine.

[7] Schlereth T., Schukraft J., Krämer-Best H.H., Geber C., Ackermann T., Birklein F. (2016). Interaction of calcitonin gene related peptide (CGRP) and substance P (SP) in human skin. Neuropeptides.

[8] Kohara H., Tajima S., Yamamoto M., Tabata Y. (2010). Angiogenesis induced by controlled release of neuropeptide substance P. Biomaterials.

[9] Ziche M., Morbidelli L., Masini E., Amerini S., Granger H.J., Maggi C.A., Geppetti P., Ledda F. (1994). Nitric oxide mediates angiogenesis *in vivo* and endothelial cell growth and migration in vitro promoted by substance P. J. Clin. Investig..

[10] Muangman P., Tamura R.N., Muffley L.A., Isik F.F., Scott J.R., Xie C., Kegel G., Sullivan S.R., Liang Z., Gibran N.S. (2009). Substance P enhances wound closure in nitric oxide synthase knockout mice. J. Surg. Res..

[11] Shi X., Wang L., Clark J.D., Kingery W.S. (2013). Keratinocytes express cytokines and nerve growth factor in response to neuropeptide activation of the ERK1/2 and JNK MAPK transcription pathways. Regul. Pept..

[12] Leal E.C., Carvalho E., Tellechea A., Kafanas A., Tecilazich F., Kearney C., Kuchibhotla S., Auster M.E., Kokkotou E., Mooney D.J. (2015). Substance P promotes wound healing in diabetes by modulating inflammation and macrophage phenotype. Am. J. Pathol..

[13] Mashaghi A., Marmalidou A., Tehrani M., Grace P.M., Pothoulakis C., Dana R. (2016). Neuropeptide substance P and the immune response. Cell. Mol. Life Sci..

[14] Słoniecka M., Le Roux S., Zhou Q., Danielson P. (2016). Substance P enhances keratocyte migration and neutrophil recruitment through interleukin-8. Mol. Pharmacol..

[15] Hong H.S., Lee J., Lee E., Kwon Y.S., Lee E., Ahn W., Jiang M.H., Kim J.C., Son Y. (2009). A new role of substance P as an injury-inducible messenger for mobilization of CD29+ stromal-like cells. Nat. Med..

[16] Hong H.S., Kim D.Y., Yoon K.J., Son Y. (2011). A new paradigm for stem cell therapy: Substance-P as a stem cell-stimulating agent. Arch. Pharm. Res..

[17] Burbach G.J., Kim K.H., Zivony A.S., Kim A., Aranda J., Wright S., Naik S.M., Caughman S.W., Ansel J.C., Armstrong C.A. (2001). The neurosensory tachykinins substance P and neurokinin A directly induce keratinocyte nerve growth factor. J. Investig. Dermatol..

[18] Hochman B., Tucci-Viegas V.M., Monteiro P.K., França J.P., Gaiba S., Ferreira L.M. (2014). The action of CGRP and SP on cultured skin fibroblasts. Cent. Eur. J. Biol..

[19] Backman L.J., Fong G., Andersson G., Scott A., Danielson P. (2011). Substance P is a mechanoresponsive, autocrine regulator of human tenocyte proliferation. PLoS ONE.

[20] Da Silva L., Carvalho E., Cruz M.T. (2010). Role of neuropeptides in skin inflammation and its involvement in diabetic wound healing. Expert Opin. Biol. Ther..

[21] Razavi R., Chan Y., Afifiyan F.N., Liu X.J., Wan X., Yantha J., Tsui H., Tang L., Tsai S., Santamaria P. (2006). TRPV1+ sensory neurons control β cell stress and islet inflammation in autoimmune diabetes. Cell.

[22] De Muckadell O.B.S., Aggestrup S., Stentoft P. (1986). Flushing and plasma substance P concentration during infusion of synthetic substance P in normal man. Scand. J. Gastroenterol..

[23] Saidi M., Kamali S., Beaudry F. (2016). Characterization of substance P processing in mouse spinal cord S9 fractions using high-resolution Quadrupole-Orbitrap mass spectrometry. Neuropeptides.

[24] Weglicki W.B., Chmielinska J.J., Tejero-Taldo I., Kramer J.H., Spurney C.F., Viswalingham K., Lu B., Tong Mak I. (2009). Neutral endopeptidase inhibition enhances substance P mediated inflammation due to hypomagnesemia. Magnes. Res..

[25] Probert L., Hanley M.R. (1987). The immunocytochemical localisation of “substance-P-degrading enzyme” within the rat spinal cord. Neurosci. Lett..

[26] Scholzen T.E., Luger T.A. (2004). Neutral endopeptidase and angiotensin-converting enzyme—Key enzymes terminating the action of neuroendocrine mediators. Exp. Dermatol..

[27] Nakamura Y., Izumi H., Fukushige R., Shimizu T., Watanabe K., Morioka N., Hama A., Takamatsu H., Nakata Y. (2015). Continuous infusion of substance P into rat striatum alleviates nociceptive behavior via phosphorylation of extracellular signal-regulated kinase 1/2. J. Neurochem..

[28] Eloy J.O., de Souza M.C., Petrilli R., Barcellos J.P.A., Lee R.J., Marchetti J.M. (2014). Liposomes as carriers of hydrophilic small molecule drugs: Strategies to enhance encapsulation and delivery. Colloids Surf. B Biointerfaces.

[29] Maitani Y., Igarashi S., Sato M., Hattori Y. (2007). Cationic liposome (DC-Chol/DOPE = 1:2) and a modified ethanol injection method to prepare liposomes, increased gene expression. Int. J. Pharm..

[30] Gibis M., Ruedt C., Weiss J. (2016). *In vitro* release of grape-seed polyphenols encapsulated from uncoated and chitosan-coated liposomes. Food Res. Int..

[31] Filipović-Grcić J., Skalko-Basnet N., Jalsenjak I. (2007). Mucoadhesive chitosan-coated liposomes: Characteristics and stability. J. Microencapsul..

[32] Tan C., Feng B., Zhang X., Xia W., Xia S. (2016). Biopolymer-coated liposomes by electrostatic adsorption of chitosan (chitosomes) as novel delivery systems for carotenoids. Food Hydrocoll..

[33] Guo J. (2003). Chitosan-coated liposomes: Characterization and interaction with leuprolide. Int. J. Pharm..

[34] Dash M., Chiellini F., Ottenbrite R.M., Chiellini E. (2011). Chitosan—A versatile semi-synthetic polymer in biomedical applications. Prog. Polym. Sci..

[35] Sorlier P., Denuzière A., Viton C., Domard A. (2001). Relation between the degree of acetylation and the electrostatic properties of chitin and chitosan. Biomacromolecules.

[36] Muzzarelli R.A. (1997). Human enzymatic activities related to the therapeutic administration of chitin derivatives. Cell. Mol. Life Sci..

[37] Menchicchi B., Fuenzalida J.P., Bobbili K.B., Hensel A., Swamy M.J., Goycoolea F.M. (2014). Structure of chitosan determines its interactions with mucin. Biomacromolecules.

[38] Menchicchi B., Fuenzalida J.P., Hensel A., Swamy M.J., David L., Rochas C., Goycoolea F.M. (2015). Biophysical analysis of the molecular interactions between polysaccharides and mucin. Biomacromolecules.

[39] Roy K., Mao H.Q., Huang S.K., Leong K.W. (1999). Oral gene delivery with chitosan—DNA nanoparticles generates immunologic protection in a murine model of peanut allergy. Nat. Med..

[40] Kong M., Chen X.G., Xing K., Park H.J. (2010). Antimicrobial properties of chitosan and mode of action: A state of the art review. Int. J. Food Microbiol..

[41] Bozzuto G., Molinari A. (2015). Liposomes as nanomedical devices. Int. J. Nanomed..

[42] Raafat D., Von Bargen K., Haas A., Sahl H.G. (2008). Insights into the mode of action of chitosan as an antibacterial compound. Appl. Environ. Microbiol..

[43] Tokura S., Ueno K., Miyazaki S., Nishi N. (1997). Molecular weight dependent antimicrobial activity by chitosan. New Macromol. Archit. Funct..

[44] Andersen T., Vanić Ž., Flaten G.E., Mattsson S., Tho I., Škalko-Basnet N. (2013). Pectosomes and chitosomes as delivery systems for metronidazole: The one-pot preparation method. Pharmaceutics.

[45] Jøraholmen M.W., Škalko-Basnet N., Acharya G., Basnet P. (2015). Resveratrol-loaded liposomes for topical treatment of the vaginal inflammation and infections. Eur. J. Pharm. Sci..

[46] Cowsik S.M., Lücke C., Rüterjans H. (1997). Lipid-induced conformation of substance P. J. Biomol. Struct. Dyn..

[47] Mertins O., Dimova R. (2011). Binding of chitosan to phospholipid vesicles studied with isothermal titration calorimetry. Langmuir.

[48] Jeon S., Yoo C.Y., Park S.N. (2015). Improved stability and skin permeability of sodium hyaluronate-chitosan multilayered liposomes by Layer-by-Layer electrostatic deposition for quercetin delivery. Colloids Surf. B Biointerfaces.

[49] Palmer T.N., Caride V.J., Caldecourt M.A., Twickler J., Abdullah V. (1984). The mechanism of liposome accumulation in infarction. Biochim. Biophys. Acta (BBA) Gen. Subj..

[50] Nevalainen T.J., Haapamäki M.M., Grönroos J.M. (2000). Roles of secretory phospholipases A2 in inflammatory diseases and trauma. Biochim. Biophys. Acta Mol. Cell Biol. Lipids.

[51] Liu J.-Y., Hu J.-H., Zhu Q.-G., Li F.-Q., Sun H.-J. (2006). Substance P receptor expression in human skin keratinocytes and fibroblasts. Br. J. Dermatol..

[52] Mitchell A.J., Lone A.M., Tinoco A.D., Saghatelian A. (2013). Proteolysis controls endogenous substance P levels. PLoS ONE.

[53] Geracioti T.D., Carpenter L.L., Owens M.J., Baker D.G., Ekhator N.N., Horn P.S., Strawn J.R., Sanacora G., Kinkead B., Price L.H. (2006). Elevated cerebrospinal fluid substance P concentrations in posttraumatic stress disorder and major depression. Am. J. Psychiatry.

[54] Zhao Y.Z., Jin R.R., Yang W., Xiang Q., Yu W.Z., Lin Q., Tian F.R., Mao K.L., Lv C.Z., Wáng Y.X.J. (2016). Using gelatin nanoparticle mediated intranasal delivery of neuropeptide substance P to enhance neuro-recovery in hemiparkinsonian rats. PLoS ONE.

[55] Wong B.J., Tublitz N.J., Minson C.T. (2005). Neurokinin-1 receptor desensitization to consecutive microdialysis infusions of substance P in human skin. J. Physiol..

[56] Sugiya H., Tennes K.A., Putney J.W. (1987). Homologous desensitization of substance-P-induced inositol polyphosphate formation in rat parotid acinar cells. Biochem. J..

[57] McConalogue K., Corvera C.U., Gamp P.D., Grady E.F., Bunnett N.W. (1998). Desensitization of the neurokinin-1 receptor (NK1-R) in neurons: Effects of substance P on the distribution of NK1-R, Galphaq/11, G-protein receptor kinase-2/3 and beta-arrestin-1/2. Mol. Biol. Cell.

[58] Holland L.N., Goldstein B.D., Aronstam R.S. (1993). Substance P receptor desensitization in the dorsal horn: possible involvement of receptor-G protein complexes. Brain Res..

[59] Kim K.-T., Kim H.-J., Cho D.-C., Bae J.-S., Park S.-W. (2015). Substance P stimulates proliferation of spinal neural stem cells in spinal cord injury via the mitogen-activated protein kinase signaling pathway. Spine J..

[60] Roggenkamp D., Köpnick S., Stäb F., Wenck H., Schmelz M., Neufang G. (2013). Epidermal nerve fibers modulate keratinocyte growth via neuropeptide signaling in an innervated skin model. J. Investig. Dermatol..

[61] Kant V., Kumar D., Prasad R., Gopal A., Pathak N.N., Kumar P., Tandan S.K. (2017). Combined effect of substance P and curcumin on cutaneous wound healing in diabetic rats. J. Surg. Res..

[62] Um J., Yu J., Park K. (2017). Substance P accelerates wound healing in type 2 diabetic mice through endothelial progenitor cell mobilization and Yes-associated protein activation. Mol. Med. Rep..

