# Supplementary material for: A Chitosan—Based Liposome Formulation Enhances the In Vitro Wound Healing Efficacy of Substance P Neuropeptide
Source: Pharmaceutics. 2017 Dec 6;9(4):56. doi: 10.3390/pharmaceutics9040056 (PMC5750662; doi:10.3390/pharmaceutics9040056)
Supplement: Supplementary file 1 [file pharmaceutics-09-00056-s001.pdf]

A Chitosan—Based Liposome Formulation Enhances the *In Vitro* Wound Healing Efficacy of Substance P Neuropeptide

Tamara Mengoni [1], Manuela Adrian [1], Susana Pereira [1], Beatriz Santos-Carballal [2], Mathias Kaiser [1] and Francisco M. Goycoolea [1,3,*]

1 Institute of Plant Biology and Biotechnology (IBBP), Westfälische Wilhelms-Universität Münster, Schlossplatz 8, 48149 Münster, Germany; tamara.mengoni@gmail.com (T.M.); m_adri02@uni-muenster.de (M.A.); ssoar_01@uni-muenster.de (S.P.); mathiaskaiser@uni-muenster.de (M.K.)
2 ChiPrO GmbH, Anne-Conway-Strasse 1, 28359 Bremen, Germany; bcarballal@chipro.de or b_sant01@uni-muenster.de
3 School of Food Science & Nutrition, University of Leeds, Leeds LS2 9JT, UK
* Correspondence: F.M.Goycoolea@leeds.ac.uk; Tel.: +44-(0)113-343-1412

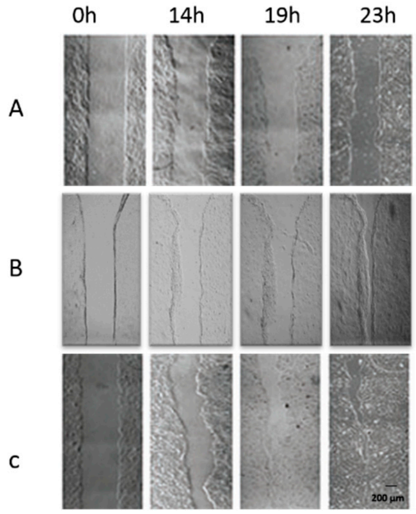

Figure S1. Exemplary pictures of time course wound healing. (**A**) shows wound progression without treatments (control), while (**B**) and (**C**) illustrate the wound progression after treatment with free SP 1×10^{-5} M and SP-CH-LP 1×10^{-5} M, respectively.
